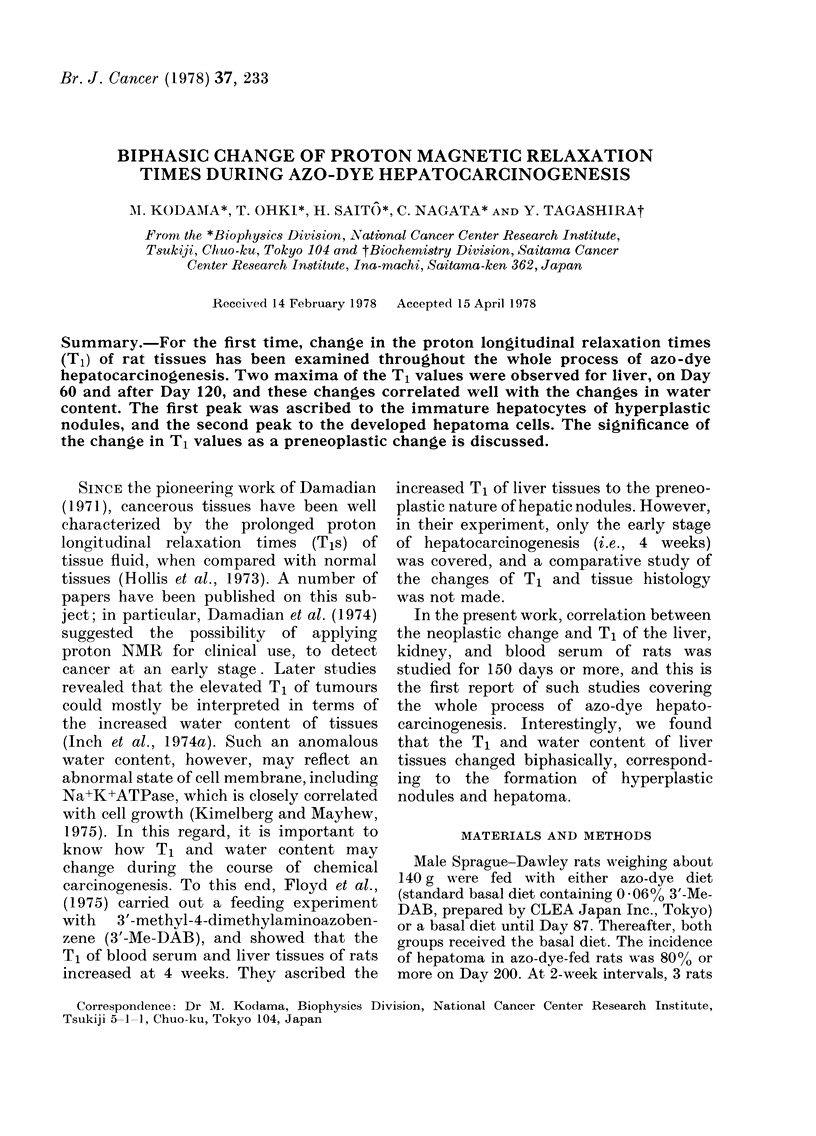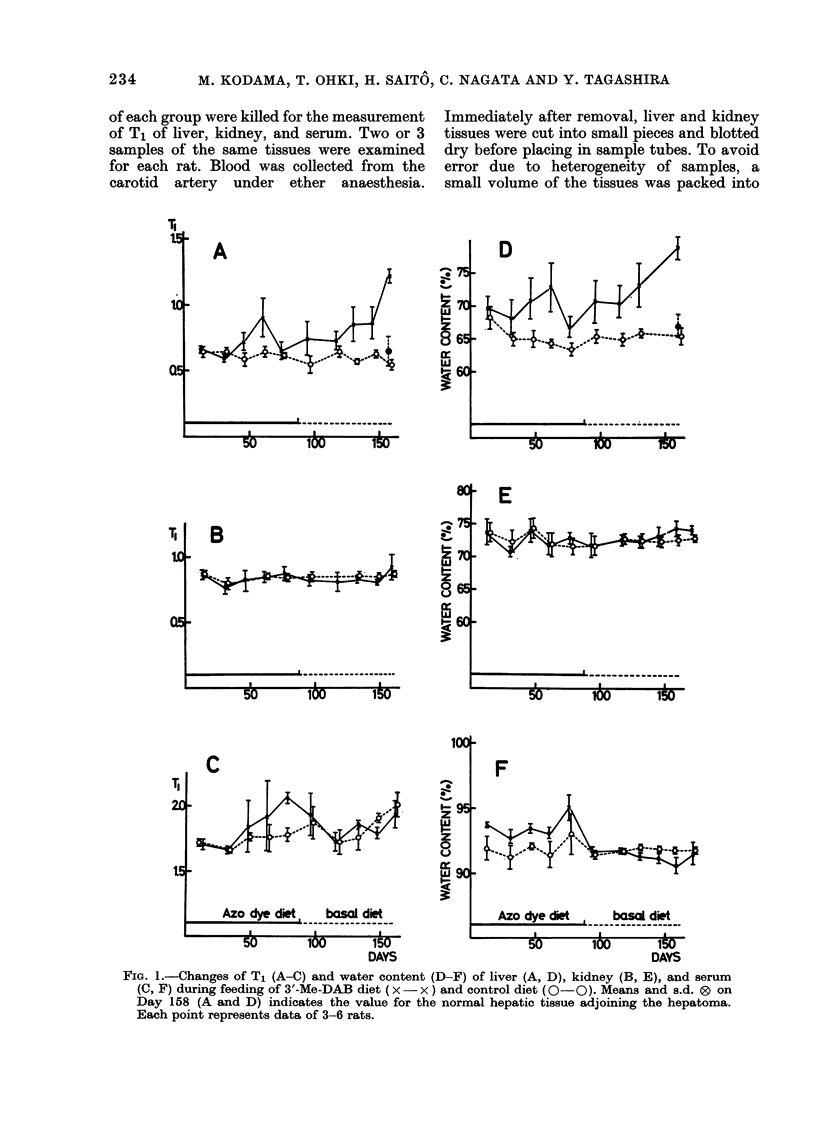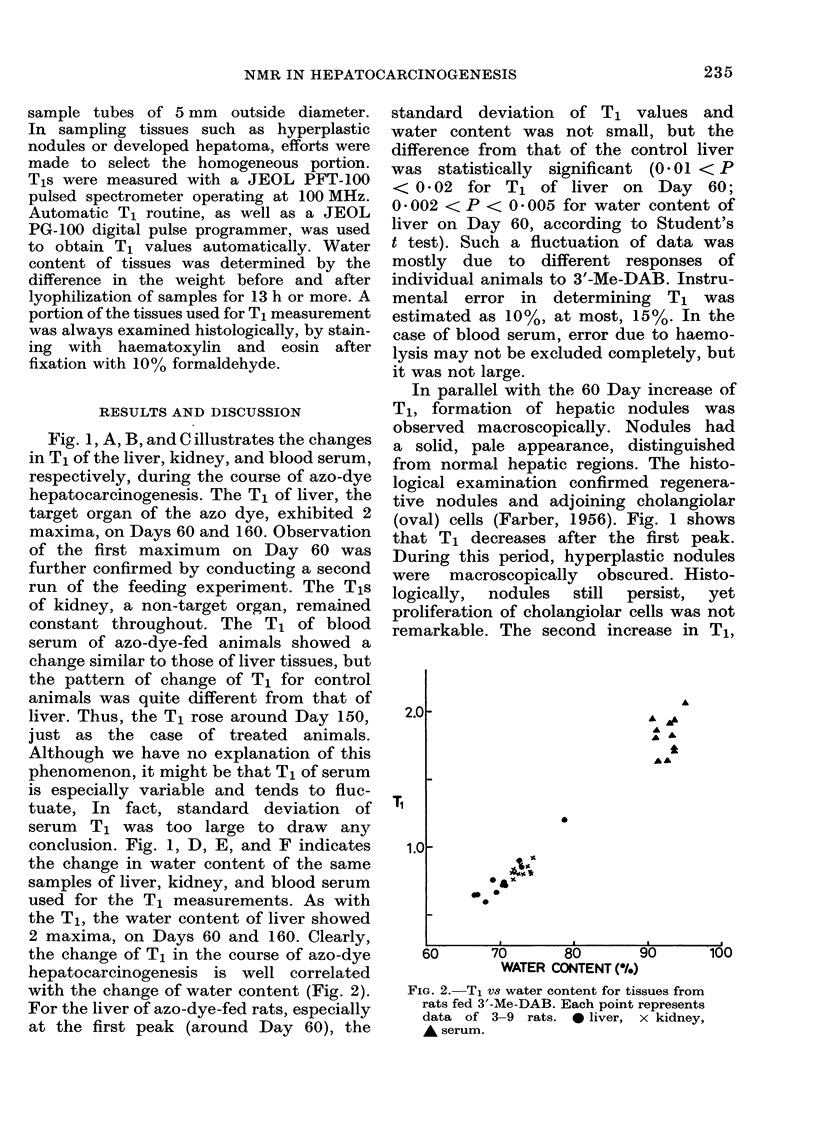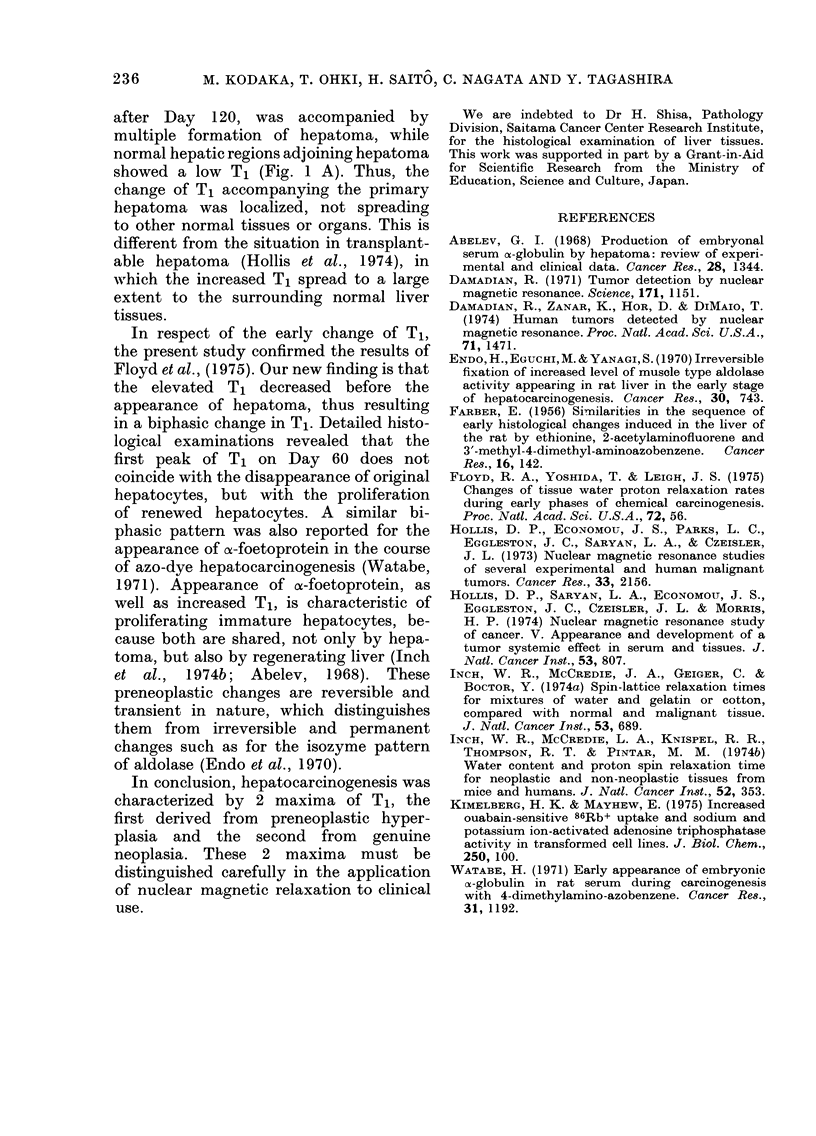# Biphasic change of proton magnetic relaxation times during azo-dye hepatocarcinogenesis.

**DOI:** 10.1038/bjc.1978.193

**Published:** 1978-08

**Authors:** M. Kodama, T. Ohki, H. Saitô, C. Nagata, Y. Tagashira

## Abstract

For the first time, change in the proton longitudinal relaxation times (T1) of rat tissues has been examined throughout the whole process of azo-dye hepatocarcinogenesis. Two maxima of the T1 values were observed for liver, on Day 60 and after Day 120, and these changes correlated well with the changes in water content. The first peak was ascribed to the immature hepatocytes of hyperplastic nodules, and the second peak to the developed hepatoma cells. The significance of the change in T1 values as a preneoplastic change is discussed.


Br. J. Cancer (1978) 37, 233

### BIPHASIC CHANGE OF PROTON MAGNETIC RELAXATION TIMES DURING AZO-DYE HEPATOCARCINOGENESIS

M. KODAMA*, T. OHKI*, H. SAITÔ*, C. NAGATA* AND Y. TAGASHIRA†

*From the *Biophysics Division, National Cancer Center Research Institute, Tsukiji, Chuo-ku, Tokyo 104 and †Biochemistry Division, Saitama Cancer Center Research Institute, Ina-machi, Saitama-ken 362, Japan*

Received 14 February 1978 Accepted 15 April 1978

**Summary.—For the first time, change in the proton longitudinal relaxation times ($T_1$) of rat tissues has been examined throughout the whole process of azo-dye hepatocarcinogenesis. Two maxima of the $T_1$ values were observed for liver, on Day 60 and after Day 120, and these changes correlated well with the changes in water content. The first peak was ascribed to the immature hepatocytes of hyperplastic nodules, and the second peak to the developed hepatoma cells. The significance of the change in $T_1$ values as a preneoplastic change is discussed.**

SINCE the pioneering work of Damadian (1971), cancerous tissues have been well characterized by the prolonged proton longitudinal relaxation times ($T_1$s) of tissue fluid, when compared with normal tissues (Hollis *et al.*, 1973). A number of papers have been published on this subject; in particular, Damadian *et al.* (1974) suggested the possibility of applying proton NMR for clinical use, to detect cancer at an early stage. Later studies revealed that the elevated $T_1$ of tumours could mostly be interpreted in terms of the increased water content of tissues (Inch *et al.*, 1974*a*). Such an anomalous water content, however, may reflect an abnormal state of cell membrane, including $Na^+K^+$ATPase, which is closely correlated with cell growth (Kimelberg and Mayhew, 1975). In this regard, it is important to know how $T_1$ and water content may change during the course of chemical carcinogenesis. To this end, Floyd *et al.*, (1975) carried out a feeding experiment with 3′-methyl-4-dimethylaminoazobenzene (3′-Me-DAB), and showed that the $T_1$ of blood serum and liver tissues of rats increased at 4 weeks. They ascribed the increased $T_1$ of liver tissues to the preneoplastic nature of hepatic nodules. However, in their experiment, only the early stage of hepatocarcinogenesis (*i.e.*, 4 weeks) was covered, and a comparative study of the changes of $T_1$ and tissue histology was not made.

In the present work, correlation between the neoplastic change and $T_1$ of the liver, kidney, and blood serum of rats was studied for 150 days or more, and this is the first report of such studies covering the whole process of azo-dye hepatocarcinogenesis. Interestingly, we found that the $T_1$ and water content of liver tissues changed biphasically, corresponding to the formation of hyperplastic nodules and hepatoma.

### MATERIALS AND METHODS

Male Sprague–Dawley rats weighing about 140 g were fed with either azo-dye diet (standard basal diet containing 0·06% 3′-Me-DAB, prepared by CLEA Japan Inc., Tokyo) or a basal diet until Day 87. Thereafter, both groups received the basal diet. The incidence of hepatoma in azo-dye-fed rats was 80% or more on Day 200. At 2-week intervals, 3 rats

Correspondence: Dr M. Kodama, Biophysics Division, National Cancer Center Research Institute, Tsukiji 5-1-1, Chuo-ku, Tokyo 104, Japan of each group were killed for the measurement of $T_1$ of liver, kidney, and serum. Two or 3 samples of the same tissues were examined for each rat. Blood was collected from the carotid artery under ether anaesthesia. Immediately after removal, liver and kidney tissues were cut into small pieces and blotted dry before placing in sample tubes. To avoid error due to heterogeneity of samples, a small volume of the tissues was packed into

FIG. 1.—Changes of $T_1$ (A–C) and water content (D–F) of liver (A, D), kidney (B, E), and serum (C, F) during feeding of 3′-Me-DAB diet (×—×) and control diet (○—○). Means and s.d. ⊗ on Day 158 (A and D) indicates the value for the normal hepatic tissue adjoining the hepatoma. Each point represents data of 3–6 rats.

sample tubes of 5 mm outside diameter. In sampling tissues such as hyperplastic nodules or developed hepatoma, efforts were made to select the homogeneous portion. $T_1$s were measured with a JEOL PFT-100 pulsed spectrometer operating at 100 MHz. Automatic $T_1$ routine, as well as a JEOL PG-100 digital pulse programmer, was used to obtain $T_1$ values automatically. Water content of tissues was determined by the difference in the weight before and after lyophilization of samples for 13 h or more. A portion of the tissues used for $T_1$ measurement was always examined histologically, by staining with haematoxylin and eosin after fixation with 10% formaldehyde.

### RESULTS AND DISCUSSION

Fig. 1, A, B, and C illustrates the changes in $T_1$ of the liver, kidney, and blood serum, respectively, during the course of azo-dye hepatocarcinogenesis. The $T_1$ of liver, the target organ of the azo dye, exhibited 2 maxima, on Days 60 and 160. Observation of the first maximum on Day 60 was further confirmed by conducting a second run of the feeding experiment. The $T_1$s of kidney, a non-target organ, remained constant throughout. The $T_1$ of blood serum of azo-dye-fed animals showed a change similar to those of liver tissues, but the pattern of change of $T_1$ for control animals was quite different from that of liver. Thus, the $T_1$ rose around Day 150, just as the case of treated animals. Although we have no explanation of this phenomenon, it might be that $T_1$ of serum is especially variable and tends to fluctuate, In fact, standard deviation of serum $T_1$ was too large to draw any conclusion. Fig. 1, D, E, and F indicates the change in water content of the same samples of liver, kidney, and blood serum used for the $T_1$ measurements. As with the $T_1$, the water content of liver showed 2 maxima, on Days 60 and 160. Clearly, the change of $T_1$ in the course of azo-dye hepatocarcinogenesis is well correlated with the change of water content (Fig. 2). For the liver of azo-dye-fed rats, especially at the first peak (around Day 60), the standard deviation of $T_1$ values and water content was not small, but the difference from that of the control liver was statistically significant ($0{\cdot}01 < P < 0{\cdot}02$ for $T_1$ of liver on Day 60; $0{\cdot}002 < P < 0{\cdot}005$ for water content of liver on Day 60, according to Student's $t$ test). Such a fluctuation of data was mostly due to different responses of individual animals to 3′-Me-DAB. Instrumental error in determining $T_1$ was estimated as 10%, at most, 15%. In the case of blood serum, error due to haemolysis may not be excluded completely, but it was not large.

In parallel with the 60 Day increase of $T_1$, formation of hepatic nodules was observed macroscopically. Nodules had a solid, pale appearance, distinguished from normal hepatic regions. The histological examination confirmed regenerative nodules and adjoining cholangiolar (oval) cells (Farber, 1956). Fig. 1 shows that $T_1$ decreases after the first peak. During this period, hyperplastic nodules were macroscopically obscured. Histologically, nodules still persist, yet proliferation of cholangiolar cells was not remarkable. The second increase in $T_1$,

FIG. 2.—$T_1$ *vs* water content for tissues from rats fed 3′-Me-DAB. Each point represents data of 3–9 rats. ● liver, × kidney, ▲ serum.

after Day 120, was accompanied by multiple formation of hepatoma, while normal hepatic regions adjoining hepatoma showed a low $T_1$ (Fig. 1 A). Thus, the change of $T_1$ accompanying the primary hepatoma was localized, not spreading to other normal tissues or organs. This is different from the situation in transplantable hepatoma (Hollis *et al.*, 1974), in which the increased $T_1$ spread to a large extent to the surrounding normal liver tissues.

In respect of the early change of $T_1$, the present study confirmed the results of Floyd *et al.*, (1975). Our new finding is that the elevated $T_1$ decreased before the appearance of hepatoma, thus resulting in a biphasic change in $T_1$. Detailed histological examinations revealed that the first peak of $T_1$ on Day 60 does not coincide with the disappearance of original hepatocytes, but with the proliferation of renewed hepatocytes. A similar biphasic pattern was also reported for the appearance of α-foetoprotein in the course of azo-dye hepatocarcinogenesis (Watabe, 1971). Appearance of α-foetoprotein, as well as increased $T_1$, is characteristic of proliferating immature hepatocytes, because both are shared, not only by hepatoma, but also by regenerating liver (Inch *et al.*, 1974*b*; Abelev, 1968). These preneoplastic changes are reversible and transient in nature, which distinguishes them from irreversible and permanent changes such as for the isozyme pattern of aldolase (Endo *et al.*, 1970).

In conclusion, hepatocarcinogenesis was characterized by 2 maxima of $T_1$, the first derived from preneoplastic hyperplasia and the second from genuine neoplasia. These 2 maxima must be distinguished carefully in the application of nuclear magnetic relaxation to clinical use.

We are indebted to Dr H. Shisa, Pathology Division, Saitama Cancer Center Research Institute, for the histological examination of liver tissues. This work was supported in part by a Grant-in-Aid for Scientific Research from the Ministry of Education, Science and Culture, Japan.

### REFERENCES

ABELEV, G. I. (1968) Production of embryonal serum α-globulin by hepatoma: review of experimental and clinical data. *Cancer Res.*, **28**, 1344.

DAMADIAN, R. (1971) Tumor detection by nuclear magnetic resonance. *Science*, **171**, 1151.

DAMADIAN, R., ZANAR, K., HOR, D. & DIMAIO, T. (1974) Human tumors detected by nuclear magnetic resonance. *Proc. Natl. Acad. Sci. U.S.A.*, **71**, 1471.

ENDO, H., EGUCHI, M. & YANAGI, S. (1970) Irreversible fixation of increased level of muscle type aldolase activity appearing in rat liver in the early stage of hepatocarcinogenesis. *Cancer Res.*, **30**, 743.

FARBER, E. (1956) Similarities in the sequence of early histological changes induced in the liver of the rat by ethionine, 2-acetylaminofluorene and 3′-methyl-4-dimethyl-aminoazobenzene. *Cancer Res.*, **16**, 142.

FLOYD, R. A., YOSHIDA, T. & LEIGH, J. S. (1975) Changes of tissue water proton relaxation rates during early phases of chemical carcinogenesis. *Proc. Natl. Acad. Sci. U.S.A.*, **72**, 56.

HOLLIS, D. P., ECONOMOU, J. S., PARKS, L. C., EGGLESTON, J. C., SARYAN, L. A., & CZEISLER, J. L. (1973) Nuclear magnetic resonance studies of several experimental and human malignant tumors. *Cancer Res.*, **33**, 2156.

HOLLIS, D. P., SARYAN, L. A., ECONOMOU, J. S., EGGLESTON, J. C., CZEISLER, J. L. & MORRIS, H. P. (1974) Nuclear magnetic resonance study of cancer. V. Appearance and development of a tumor systemic effect in serum and tissues. *J. Natl. Cancer Inst.*, **53**, 807.

INCH, W. R., MCCREDIE, J. A., GEIGER, C. & BOCTOR, Y. (1974*a*) Spin-lattice relaxation times for mixtures of water and gelatin or cotton, compared with normal and malignant tissue. *J. Natl. Cancer Inst.*, **53**, 689.

INCH, W. R., MCCREDIE, L. A., KNISPEL, R. R., THOMPSON, R. T. & PINTAR, M. M. (1974*b*) Water content and proton spin relaxation time for neoplastic and non-neoplastic tissues from mice and humans. *J. Natl. Cancer Inst.*, **52**, 353.

KIMELBERG, H. K. & MAYHEW, E. (1975) Increased ouabain-sensitive $^{86}Rb^+$ uptake and sodium and potassium ion-activated adenosine triphosphatase activity in transformed cell lines. *J. Biol. Chem.*, **250**, 100.

WATABE, H. (1971) Early appearance of embryonic α-globulin in rat serum during carcinogenesis with 4-dimethylamino-azobenzene. *Cancer Res.*, **31**, 1192.